# Excess cases of influenza-like illnesses synchronous with coronavirus disease (COVID-19) epidemic, France, March 2020

**DOI:** 10.2807/1560-7917.ES.2020.25.14.2000326

**Published:** 2020-04-09

**Authors:** Pierre-Yves Boëlle, Cécile Souty, Titouan Launay, Caroline Guerrisi, Clément Turbelin, Sylvie Behillil, Vincent Enouf, Chiara Poletto, Bruno Lina, Sylvie van der Werf, Daniel Lévy-Bruhl, Vittoria Colizza, Thomas Hanslik, Thierry Blanchon

**Affiliations:** 1 Sorbonne Université, Institut Pierre Louis d’Epidemiologie et de Santé Publique, Paris, France; 2 INSERM, Institut Pierre Louis d’Epidemiologie et de Santé Publique, Paris, France; 3 Santé Publique France, Saint Maurice, France; 4 Institut Pasteur, Unité de Génétique Moléculaire des Virus à ARN, Paris, France; 5 Institut Pasteur, Centre Coordonnateur du Centre National de Référence des virus des infections respiratoires (dont la grippe), Paris, France; 6 UMR CNRS 3569, Paris, France; 7 Université Paris Diderot, Sorbonne Paris Cité, Unité de Génétique Moléculaire des Virus à ARN, Paris, France; 8 Laboratoire de Virologie, Hospices Civils de Lyon, Institut des Agents Infectieux (IAI), Centre National de Référence des virus respiratoires (dont la grippe), Centre de Biologie et de Pathologie Nord, Groupement Hospitalier Nord, Lyon, France; 9 Université de Lyon, Virpath, CIRI, INSERM U1111, CNRS UMR5308, ENS Lyon, Université Claude Bernard Lyon 1, Lyon, France

**Keywords:** COVID-19, Surveillance Sentinel Network, influenza-like illness

## Abstract

Several French regions where coronavirus disease (COVID-19) has been reported currently show a renewed increase in ILI cases in the general practice-based *Sentinelles* network. We computed the number of excess cases by region from 24 February to 8 March 2020 and found a correlation with the number of reported COVID-19 cases so far. The data suggest larger circulation of severe acute respiratory syndrome coronavirus 2 (SARS-CoV-2) in the French population than apparent from confirmed cases.

The *Sentinelles* network monitors influenza-like illnesses (ILI) and acute respiratory infections (ARI) in general practice in France [1]. The 2019/20 influenza epidemic reached its peak in mid-February in France [2] and it was expected that ILI incidence would decrease thereafter. However, in the first week of March 2020, we observed a renewed increase in ILI cases in some regions. Several nasopharyngeal swabs collected in that period by *Sentinelles* general practitioners (GPs) tested positive for severe acute respiratory syndrome coronavirus 2 (SARS-CoV-2). Here, we quantified the number of consultations for ILI in France in early March 2020 in excess of what was expected to be caused by the influenza epidemic. As coronavirus disease (COVID-19) cases were first identified in France at the end of January 2020 [3] we examined the relationship between ILI reports and reported cases of COVID-19.

## Sentinelles reporting of influenza-like illness

Cases of ILI and ARI are reported in real time by participating GPs (ca 600 GPs) all over France. The case definition for ILI is fever of sudden onset (> 39 °C) with respiratory signs (cough, running nose) and myalgia in a person of any age. Cases of ARI include any disease with respiratory signs and are only monitored in people older than 65 years, unlike ILI. In addition, *Sentinelles* GPs take nasopharyngeal swabs in one ILI case and up to two ARI cases per week for viral characterisation [4]. Influenza virus (see Supplement), respiratory syncytial virus, human rhinovirus and human metapneumovirus are routinely looked for, and SARS-CoV-2 was added at the end of February 2020. In the first week of testing, week 9 (the week starting 24 February 2020), none of 119 submitted swabs tested positive for SARS-CoV-2. In week 10 of 2020 (the week starting 2 March 2020), 93 swabs were collected in ILI patients and 23 in ARI patients, and two swabs in each category were positive for SARS-CoV-2. 

For ILI, one of the two positive ILI cases had been collected because the patient reported a direct link with an existing cluster in the east of France. We computed the expected number of ILI consultations using the superposition of a seasonal [5] and an epidemic influenza component [6], as detailed in the Supplement. Excess cases were computed as the difference between observed cases and expected numbers. This modelling approach allowed computing excess cases in 11 of 13 regions but did not provide a good fit in the remaining two regions where a marked influenza peak was not present (see Supplement). The overall ILI incidence showed renewed increase with 33 (95% credible interval (CrI): −8 to 64) consultations per 100,000 in excess during week 9 and with 84 (95% CrI: 447 to 108) consultations per 100,000 in excess in week 10. Four of the 11 regions displayed positive excess (CrI excluding 0) in week 9 and seven regions in week 10 (Figure).

**Figure:**
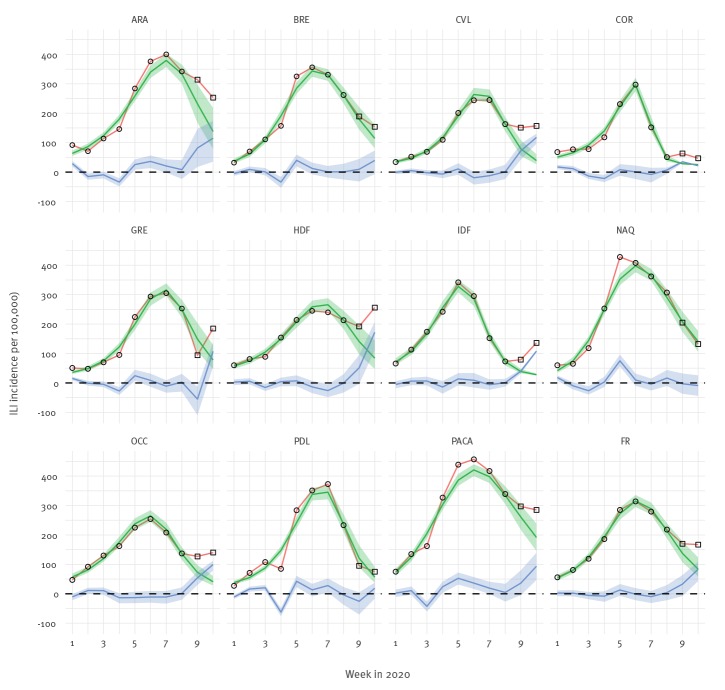
Number of consultations for influenza-like illness (per 100,000) in France and 11 French regions, with expected number of consultations fitted on the first eight weeks, week 1–10, 2020 (n = 11 regions)

ARA: Auvergne-Rhone-Alpes; BRE: Brittany; CVL: Centre-Val de Loire; COR: Corsica; GRE: Grand-Est; HDF: Hauts de France; IDF: Ile de France; ILI: influenza-like illness; NAQ: Nouvelle Aquitaine; OCC: Occitanie; PACA: Provence-Alpes-Cote d’Azur; PDL: Pays de la Loire.

Blue: excess consultations, computed by difference between observations (squares) and expected counts. Green: expected number of consultations fitted on the first eight weeks of 2020 (circles).

Confirmed COVID-19 case counts were obtained from the Santé Publique France website [7]. The number of excess cases correlated with the cumulated number of COVID-19 cases reported in the same regions (r = 0.59; p < 0.05) in week 10. Assuming exponential growth (typical of early epidemics) from week 8 to 10, 2020, we found that the excess number of cases had an exponential growth rate per week of 0.69 (95% CrI: 0.55–0.86) in Grand-Est (GRE), 0.67 (95% CrI: 0.55 to 0.83) in Ile de France (IDF), 0.68 (95% CrI: 0.56 to 0.83) in Hauts de France (HDF), 0.61 (95% CrI: 0.48 to 0.75) in Occitanie (OCC) and 0.56 (95% CrI: −1.6 to 0.8) in France overall (Table).

**Table:** Excess consultations per 100,000 by region and cumulative number of confirmed coronavirus disease (COVID-19) cases in the corresponding weeks, France, week 9–10, 2020 (n = 11 regions)

Region	Consultations in excess/100,000)		Confirmed COVID-19 cases (cumulative)		Population (in millions)
	Week 9	Week 10	Week 9	Week 10	
Auvergne-Rhone-Alpes (ARA)	82 (17 to147)	115 (36 to 174)	28	146	8
Brittany (BRE)	9 (−32 to 44)	40 (−7 to 73)	5	59	3.3
Centre-Val de Loire (CVL)	72 (43 to 96)	117 (97 to 130)	0	17	2.6
Corsica (COR)	35 (32 to 37)	21 (20 to 23)	0	5	0.3
Grand-Est (GRE)	−55 (−109 to −13)	107 (56 to 138)	5	259	5.5
Hauts de France (HDF)	51 (−3 to 94)	172 (111 to 208)	34	163	6.0
Ile de France (IDF)	40 (32 to 46)	107 (103 ti 110)	18	191	12.3
Nouvelle Aquitaine (NAQ)	−3 (−110 to 32)	−9 (−44 to 26)	4	28	6.0
Occitanie (OCC)	54 (30 to75)	99 (79 to 112)	4	37	5.9
Pays de la Loire (PDL)	−25 (−70 to 5)	18 (−16 to 38)	4	25	3.5
Provence-Alpes-Cote d’Azur (PACA)	36 (−4 to 80)	94 (47 to 140)	8	52	5.0
**France **	**33 (−8 to 64)**	**84 (44 to 108)**	**110**	**982**	**64.9^a^**

^a^ Total population of all 13 regions of mainland France.

For ARI, the two positive swabs had been collected in the regions Bourgogne-Franche Comté (BFC) and GRE, from a total of seven swabs in these two regions. We estimated that 760 (95% CrI: 219–1,706) ARI consultations in those older than 65 years in these two regions (BFC and GRE) could have been caused by COVID-19 during week 10 (2 in 7 for a total of 2,600 ARI visits). We did not pursue modelling excess cases for ARI as the time series (shown in the Supplement) were too noisy to properly break down the data into an expected seasonal part plus excess cases. 

## Discussion

This is the first time that an increase in ILI cases was observed simultaneously in several regions after the peak of the annual influenza epidemic in the last 30 seasons of routine surveillance in France with the *Sentinelles* network [9]. Several processes may have contributed to this observation, including, among others, characteristics of the influenza season, change in population behaviour or increase in COVID-19 incidence.

Influenza A (H1N1)pdm09 and influenza B viruses co-circulated in 2019/20 in France [10] (see Supplement). Seasons with type B virus circulation can lead to a slow decay in incidence but a renewed increase is atypical [1]. The 2-week-long normal school holidays started between mid-February and early March depending on the region. While holidays may change the dynamics of influenza [9], this generally has an effect in the ascending phase of the epidemic rather than after the peak.

The COVID-19 pandemic may have changed the health-seeking behaviour of patients and, to some extent, the reporting of the contributing GPs although they conform to a case definition. In the crowdsourced surveillance system grippenet.fr [10], there was no evidence of increasing consultations: 30% consulted with a GP in for ILI during week 10, compared with 38% in week 9. As information regarding the coronavirus risk is widespread, a uniform increase in all regions could have been expected in this scenario rather than only in a few regions. We however acknowledge that increased consultation rates in regions where COVID-19 is the most reported may be possible.

The similarity between COVID-19 and influenza symptoms makes it possible that the excess ILI cases were due to COVID-19 cases. The presence of SARS-CoV-2-positive swabs in the patients supports this possibility. The correlation between the number of confirmed cases and the computed excess is also consistent with this scenario. In this case, the excess in cases is compatible with an exponential growth at an estimated rate of ca 0.7 per week. Interestingly, this estimate was very similar in the four most affected regions (GRE, IDF, OCC, HDF). It may prove useful to help calibrate models for studying the impact of the COVID-19 pandemic.

Estimating the true number of COVID-19 cases can only be tentative at this early stage of the epidemic with data from a non-dedicated surveillance system. It is likely that the case definition of ILI does not allow identifying all COVID-19 cases. In addition, only a few cases were virologically tested, adding uncertainty in the estimates. Yet, the rate of excess ILI consultations applied to the French regions yielded a number of cases far in excess of confirmed cases, with 12,840 cases in IDF and around 10,000 cases in the regions GRE and HDF. Likewise, the extrapolated number of COVID-19 presenting as ARI was 760 in those older than 65 years in the two eastern regions (GRE and BFC): this was already twice the number of reported cases in these regions at the time (n = 379), all ages combined. 

As we enter a period of generalised circulation of SARS-CoV-2, surveillance based on clinical description and swabbing by GPs will prove essential in assessing the situation.

## Supplementary Data

372000 Supplement
