# Supplementary material for: Excess cases of influenza-like illnesses synchronous with coronavirus disease (COVID-19) epidemic, France, March 2020
Source: Euro Surveill. 2020 Apr 9;25(14):2000326. doi: 10.2807/1560-7917.ES.2020.25.14.2000326 (PMC7160441; doi:10.2807/1560-7917.ES.2020.25.14.2000326)
Supplement: Supplement [file 20-00326_BOELLE_Supplement.pdf]

Supplementary material

"This supplementary material is hosted by Eurosurveillance as supporting information alongside the article [Excess cases of influenza-like illnesses synchronous with coronavirus disease (COVID-19) epidemic, France, March 2020], on behalf of the authors, who remain responsible for the accuracy and appropriateness of the content. The same standards for ethics, copyright, attributions and permissions as for the article apply. Supplements are not edited by Eurosurveillance and the journal is not responsible for the maintenance of any links or email addresses provided therein.

The number of expected ILI consultations reported to the *Sentinelles* network was modelled as the superposition of 2 components: an "epidemic" component due to influenza infections and a background seasonal signal not due to influenza (4). More precisely, we fitted the influenza epidemic part using Richard's model(5). Richard's model is a 4 parameters equation describing the cumulated number of cases at time *t* as follows :

$$I_R(t) = A\left(1 + \nu\, exp\left(1 + \nu + \frac{\mu}{A}(1+\nu)^{1+1/\nu}(\lambda - t)\right)\right)^{-1}$$

The seasonal part was modelled as

$$S(t) = B0 + B1\left(1 + \cos\left(\frac{2\pi}{T}t + \varphi\right)\right)$$

We fitted this model assuming that the number of ILI consultations ILI(t) was Poisson distributed with mean $I_R(t) + S(t)$ over the 8 first weeks of 2020 and used non-epidemic periods in years 2016 to 2020 additional information to estimate $S(t)$ (see appendix for more detail). Excess ILI cases was defined as observation minus the expected part, i.e. $E(t) = ILI(t) - I_R(t) - S(t)$.

In a second step, we fitted excess cases by an exponential increase $C_E(t) = C0\,\exp(r\,t)$, and assumed that number of ILI cases for weeks 8 to 10 was Poisson distributed with mean $I_R(t) + S(t) + C_E(t)$. We tied the number of excess cases at 0 in week 1 of 2020 for this estimation.

Estimations were performed with STAN. Code is available upon request.

Example data used for estimating excess cases in a French region. The orange points were used to estimate the seasonal part; the first peak in the black part was fit using Richard's model plus the seasonal part, and excess was computed over the last 2 weeks as indicated in figure S3.

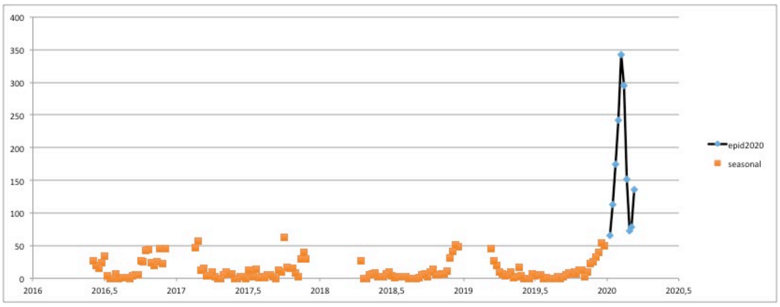

Figure S1: Example data used for excess consultations modelling. The black line corresponds with the 2020 epidemic. The orange points corresponds with the seasonal background information from previous years.

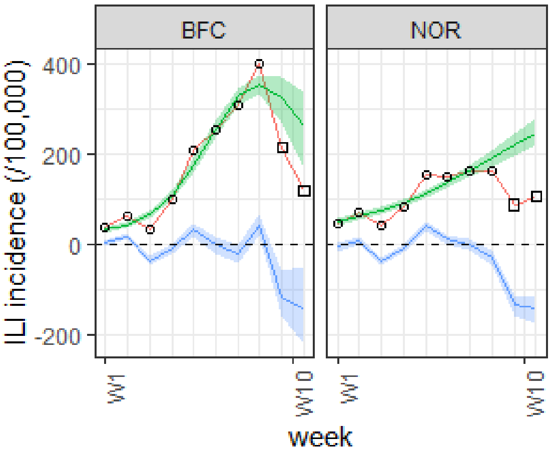

Figure S2 : Two French regions where the model failed to describe the data.

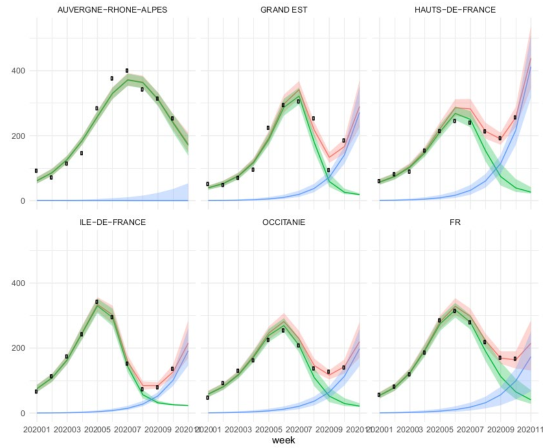

Figure S3 : Decomposition of observed ILI cases in France (dots) in a seasonal + epidemic part (green) and excess part (blue). The red curve is the sum of the two components.

Virological data from ILI surveillance

Figure S4 shows the results of virological detection for the flu during the 2019-2020 season. (http://www.sentiweb.fr/france/fr/?page=bulletin) Collection of data was interrupted in week 11 as GPs were not routinely equipped with masks. It started anew in week 12 and 13.

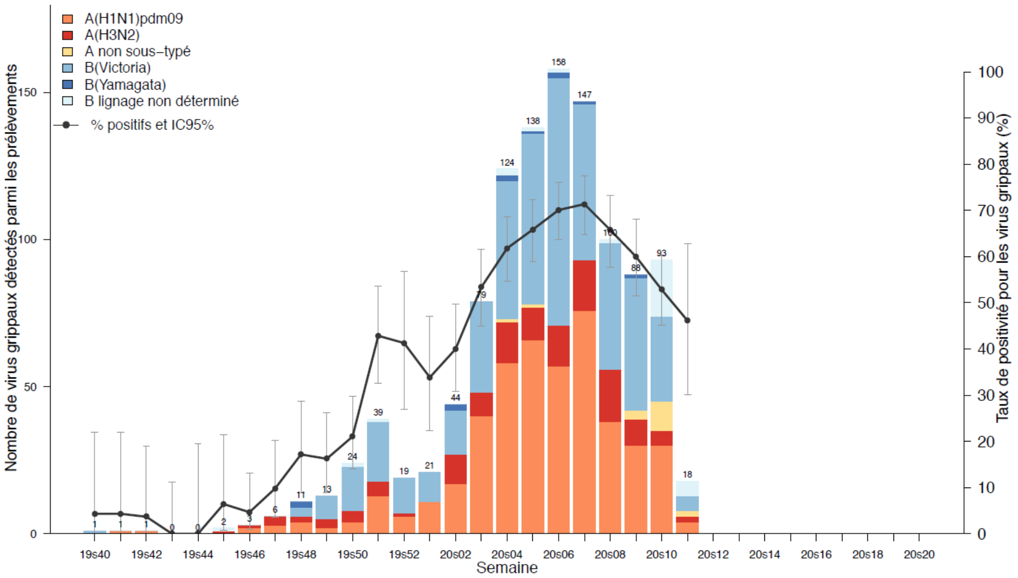

Figure S4 : Virological data collected during ILI surveillance in 2019-2020.

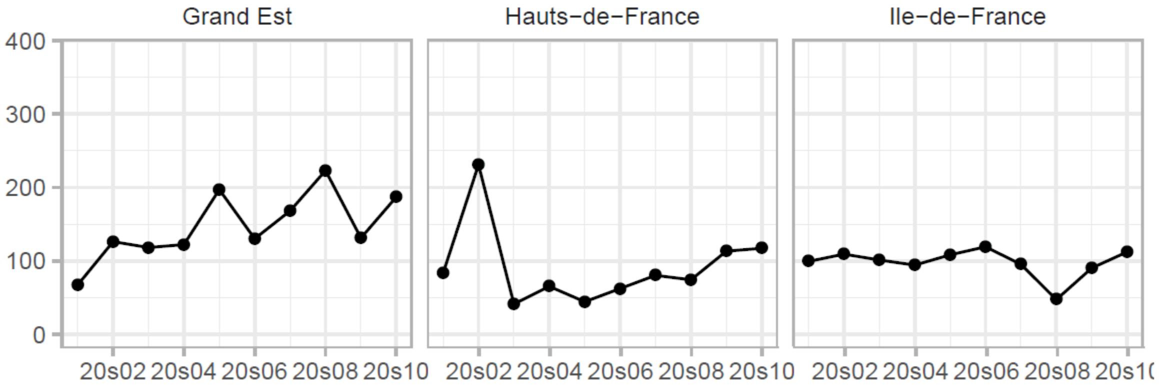

Figure S5 : IRA incidence (/100000) in the 65 years-old in the 3 regions where the increase in COVID19 cases was the largest.
